# French database of children and adolescents with Prader-Willi syndrome

**DOI:** 10.1186/1471-2350-9-89

**Published:** 2008-10-02

**Authors:** Catherine Molinas, Laurent Cazals, Gwenaelle Diene, Melanie Glattard, Catherine Arnaud, Maithe Tauber, French Reference Centre for PWS (FrRefC-PWS)

**Affiliations:** 1 Centre de Référence du syndrome de Prader-Willi, Division of Endocrinology, Genetics, Gynaecology and Bone Diseases, Hôpital des Enfants, Toulouse, France; 2 Division of Diabetology, Metabolic Diseases and Nutrition, Hôpital Rangueil, Toulouse, France; 3 National Institute of Health and Medical Research INSERM U558, University Paul Sabatier, Toulouse, France; 4 Unit of Clinical Research, Hospital Toulouse, France

## Abstract

**Background:**

Prader-Willi syndrome (PWS) is a rare multisystem genetic disease leading to severe complications mainly related to obesity. We strongly lack information on the natural history of this complex disease and on what factors are involved in its evolution and its outcome. One of the objectives of the French reference centre for Prader-Willi syndrome set-up in 2004 was to set-up a database in order to make the inventory of Prader-Willi syndrome cases and initiate a national cohort study in the area covered by the centre.

**Description:**

the database includes medical data of children and adolescents with Prader-Willi syndrome, details about their management, socio-demographic data on their families, psychological data and quality of life of the parents. The tools and organisation used to ensure data collection and data quality in respect of good clinical practice procedures are discussed, and main characteristics of our Prader-Willi population at inclusion are presented.

**Conclusion:**

this database covering all the aspects of PWS clinical, psychological and social profiles, including familial psychological and quality of life will be a powerful tool for retrospective studies concerning this complex and multi factorial disease and could be a basis for the design of future prospective multicentric studies. The complete database and the Stata.do files are available to any researcher wishing to use them for non-commercial purposes and can be provided upon request to the corresponding author.

## Background

Prader-Willi syndrome (PWS) is a rare genetic neurodevelopmental disorder related to the lack of expression of paternal genes in the q11-q13 region of chromosome 15 first described in 1956 [1]. The incidence of PWS is about one out of 25000 births [2-4], most cases are sporadic and familial cases are rare. PWS infants exhibit severe hypotonia already present at birth that partially improves, explaining in part suckling and swallowing troubles, failure to thrive (i.e. failure to get weight besides normal or increased caloric intake) as well as delayed psycho-motor development [5-7]. After this initial phase of about 2 years, the most striking sign appears: an early and severe obesity related to an increasing appetite and the decrease of satiety with an overwhelming eating obsession [8]. Furthermore, a wide range of associated troubles is reported in children, adolescents and adults: low and delayed motor and oral-motor skills, learning disabilities, growth hormone and sex hormones deficiencies probably due to an unknown hypothalamic dysfunction and resulting in short stature, increased body fat with a decreased lean mass, cryptorchidism and absence or blunted puberty [8]. Other common concerns include strabismus, scoliosis, osteoporosis, type 2 diabetes mellitus, hypertension, hypoventilation, heart failure, skin problems, sleep disturbances and skin picking (sometimes severe). Early diagnosis confirmed by genetic analysis combined with multidisciplinary care including hormonal therapies can dramatically smooth over PWS troubles and improve the quality of life of these patients and their family [9-13]. However, the constant and long-term need for food restriction, behaviour management and medical care may be stressful for PWS patients and family members [14,15].

In 2003, the French Ministry of Health launched a strategic plan to optimize the diagnosis and the care of people with rare disorders and strongly encouraged the setting-up of reference centres for rare diseases. Since the late 90's, French teams involved in PWS care had initiated collaborations to share clinical knowledge and experience and in 2004, the French Ministry of Health agreed to approve a reference centre for Prader-Willi syndrome, covering the southern half of the country. In 2007 the reference centre was asked to extend to the whole country with the help of the French family association. PWS reference centre, as other reference centres, has three main goals: optimize patient's diagnosis, care and management throughout life, inform and train health professionals and develop and/or support clinical, epidemiological and basic research projects. The aim of this article is to describe the database constructed to reach one of the first objectives of PWS reference centre: to take inventory of PWS cases and initiate a cohort study in the area covered by the centre. This database will be of primary interest to improve the knowledge of PWS disease: it will be a powerful tool for future specific research projects such as epidemiological studies. We strongly lack information on the natural history of this severe disease, on factors involved in its evolution and on the outcomes of these children. There are few cohort studies [16-19] and few data related to overall PWS patients and family's cares are available. To our knowledge, there is no published database covering all the aspects of PWS clinical, psychological and social profiles, including familial psychological, quality of life and type of care. Data collected could be added to the European database in development [20] as our team participated to the French group of the European database.

## Construction and content

Our objective was to describe in a cross sectional approach clinical and psychological characteristics of children and adolescents with PWS, their management and follow-up as well as their parents' psychological profile and quality of life. Searches of PWS physicians' medical records, PWS patients association listing and genetic diagnosis laboratory databases allowed us to estimate the total eligible PWS patients, about 180 in the reference centre area in 2004. Based on the estimated prevalence of the disease ranging from 1/25 000 to 1/30 000 and a total population of 260 000 births per year in the area, the theoretical calculated number fell between 164 and 198 children and adolescents. All of the paediatric endocrinologists (public and private) taking care of PWS patients, epidemiologists and psychologists of the reference centre have been involved in study design, forms building and validation processes to ensure data collection feasibility and data quality. This first step was of primary interest because the huge amount of data that could be gathered from these PWS patients presenting severe multiple pathologies had to be sorted out and simplified in order to obtain standardized questionnaires covering most of the components of this complex disease and adapted to physicians' work environment and habits. Questionnaires' design has been discussed during five meetings and was completed in 1 year. Consensus has been reach on i) a baseline form including Medical and Socio-Demographic data (MSD form) filled in by patient's physician and a familial psychological and quality of life form filled in by the parents ii) a yearly follow-up form including medical and psychological data of the patients iii) this questionnaire will be subsequently modified and adapted to adult patients.

### Medical and socio-demographic data at inclusion

MSD form is a 265-item paper questionnaire designed to be filled in by each PWS patient's physician, according to clinical examination and medical records information. When completed, MSD form is mailed to the coordination team and data are entered into the database by a Clinical Research Associate (CRA). Variables of the MSD form are summarized in Table 1.

**Table 1:** List of variables of the medical and socio-demographic (MSD) form

**Domain**	**Number of variables recorded**
Identification of the patient	11
Genetic diagnosis	4
Comprehensive care	2
Medical history of the family	16
Parents data	8
Familial situation	6
Professional situation of the parents	10
Social status	3
Adhesion to the parents' association	3
Pregnancy	10
Birth	13
Auxological measurements	19
Endocrine dysfunctions:	
- GH deficiency	12
- GH treatment	12
- Hypogonadism	7
- Cryptorchidism	4
- Hypothyroidism	6
Psychomotor development and language	20
Management:	
- Nutritional management	4
- Physiotherapy	4
- Speech and language management	4
- Psychomotricity	4
- Ophthalmologic disorders	3
- ENT	4
- Other specific management	4
Behavioural troubles	32
Medical Complications	27

Mean time length required to complete this first MSD form has been estimated to be 60 min (30 min during medical consultation and 30 min dedicated to source data collection). As in some cases PWS medical records may require a long time to be inspected, five of the nine centres required CRA assistance to fill in clinical data questionnaires.

### Familial psychological data at inclusion

Familial psychological data were gathered using 3 different paper forms, given and explained at a routine visit, and filled in by the parents at home. In total, time length required is about 45 to 60 min.

The first form corresponds to the Child Behaviour CheckList questionnaire (CBCL), a well validated instrument designed to assess parent-reported behavioural and emotional problems [21-24]. It is appropriate for use in 1.5 to 18 years old- children and adolescent populations and has been widely used in Prader Willi studies [25-28]. We asked both parents to fill the CBCL together unless it was impossible for familial reasons. CBCL questionnaires have been computed using "Assessment Data Manager, version 4.0" software to compute raw scores, T-scores and clinical translated scores (clinical-borderline-normal) [21,22].

The second form contains three validated scales evaluating parent-reported stress, anxiety and depression. Each parent has to complete his/her own questionnaire separately.

- the 14-item Perceived Stress Scale (PSS14) [29] is validated to measure last month's sensed stress. It is scaled in a positive direction with a higher score denoting higher stress. PSS-14 has been widely used in psychological studies due to its psychometrical properties [30].

- Spielberger Test Anxiety Inventory (STAI) (Y form, 20 items) [31] measures trait-anxiety (trait-anxiety is a personality feature, a usual and stable anxiety trend). It is scaled in a positive direction and raw scores are transformed in T-scores according to French norms [32]. STAI is widely used in psychological studies and has good psychometrical properties.

- Beck Depression Inventory-II (BDI-II) is a 13-item scale [33] which allows depression severity measurement as well as nervous depression versus anxiety differentiation. The BDI-II is one of the most widely accepted clinical instruments [30,34]. Higher scores indicate greater depressive symptomatology. Participants with scores in the moderate-to-severe range, suggesting clinically significant levels of depressive symptoms, were identified by the psychologist of the reference centre; patient's physician was informed and offered assistance to obtain further assessment and treatment. This procedure is outlined to families at the time the familial psychological questionnaires are given to them by the physician.

The third form is the 1997 World Health Organisation Quality of Life Questionnaire (WHOQOL-BREF). This 26-item questionnaire is a sound, cross-culturally valid assessment of quality of life (QOL), as reflected by the four domains explored (physical health, psychological health, social relationships and environment) and its two individually scored items about an individual's overall perception of QOL and health. The four domains and the two individual items are scaled in a positive direction with higher scores denoting higher QOL. WHOQOL-BREF must be filled in by each parent. This questionnaire has been widely and internationally used, translated into French and validated, and reported to be sensitive, accurate and homogeneous [35].

The coordination centre in Toulouse conducted a pilot study involving 12 Prader-Willi's families in order to confirm the feasibility of a familial psychological study. Families first received an explanation from the psychologist, filled in the questionnaires at home and mailed them to the coordination team of the reference centre (prepaid envelops). In total, 18 out of 24 questionnaires (75%) were returned in 4 weeks. None of the parents reported difficulties about understanding and/or answering the whole questionnaire, nor psychological difficulties related to BDI-II questionnaire. We did not perform a statistical analysis on these questionnaires.

### Follow-up form

A yearly follow-up paper form has to be completed to supply the longitudinal part of the study. It is filled in by investigators according to patient's clinical examination and medical records information. When completed, the follow-up form is mailed to the coordination team and data are entered into the database by a CRA. Variables of the MSD follow-up form are summarized in Table 2. Mean time length required to complete the follow-up form has been estimated to 30 min (15 min during medical consultation and 15 min dedicated to source data collection).

**Table 2:** List of variables of the follow-up medical and socio-demographic form (follow-up MSD form)

**Variables of the follow-up medical and socio-demographic form (follow-up MSD form)**
**Patient identification**
- Identification number, date, date of initial visit
- Changes in familial medical history and familial situation since previous visit
**Auxological and clinical measurements**
Height, weight, BMI, head circumference, waist, hip, pubertal stage, pulse, blood pressure, right- or left-handed
**Medical complications, operations since previous visit**
**Psychomotor development**
schoolar, extra-schoolar activities
**Treatments**
GH, treatment for hypogonadism, hypothyroidism, other
**Management**
Nutritional management, physiotherapy, speech and language management, psychomotricity, ophthalmology, orthopaedic, psychological management, psychiatric management, other specific management
**Behavioural troubles**
IQ, Food-related troubles, anxiety, depression, obsessive compulsive disorder, psychotic aspects
**Biological data**
**Imaging data**

The follow-up form includes a psychological questionnaire evaluating the overall psychopathology of patients and filled in by the parents. This questionnaire has not been validated yet but is routinely used for adults with PWS by the coordination centre, which has been involved in the care of adult PWS patients for many years [36]. We chose this specific questionnaire in order to evaluate the evolution of psychological troubles from childhood to adulthood. Mean time to complete this questionnaire is about 10 minutes.

## Construction

Data entry is done using Microsoft^® ^Access 2003 (Microsoft Corp, Redmond, WA 98052-6399, USA). Database structure is as follows: Main table contains administrative data plus MSD form items. For each of the three psychological and QOL questionnaires, scores are stored in a table linked one-to-many to the main table. One table linked one-to-many to the main table contains annual follow-up items. A user friendly interface including rolling menus and data entry forms has been built to allow database use by unskilled users. In data entry forms, fields are organized in regard to paper forms presentation and include limits which where applicable to minimize the risk of error during the data entry process.

A set of Stata 8.2 programs (StataCorp, College Station, Texas 77845, USA) has been developed to quicken and secure data management procedures prior to statistical analyses. Briefly, all raw tables are match-merged, variables are labelled, recoded when needed and calculated variables are computed to generate a Stata file containing the whole study data. This procedure is completed with a set of calculated coherence controls. Birth weight and birth length are expressed in Standard Deviation (SD) according to Usher and Mc Lean tables [37], BMI Z-scores are calculated and obesity defined as BMI > 97th centile, according to French reference curves for BMI [38].

Computing time required for this complete procedure is about five minutes and the entire procedure can be done by unskilled users, simply clicking on Stata.do files icons. All calculations are recorded in a log file to ensure computation traceability, and log files are archived with main data and results files every time a statistical analysis is performed.

## Data collection

Database and data collection have been approved by the 2 French national data protection authorities, the "Comité Consultatif sur le Traitement de l'Information en matière de Recherche dans le domaine de la Santé" (Advisory council for treatment of information in terms of healthcare research) and the "Commission Nationale de l'Informatique et des Libertés" (French Data Protection Authority). We got the approval of the president of the local ethic committee (CPP 1 Toulouse, Dr H. Grandjean). Data collection is monitored by CRA in respect of Good Clinical Practice (ICH-GCP) procedures. All patients with Prader-Willi syndrome aged 0 to 20 living in the region covered by the reference centre are eligible to participate. Exclusion criterion is lack of confirmation of PWS by genetic diagnosis. The study is presented to all eligible patients by their physician during a medical consultation. After the informed written consent is obtained from the patient and his family, investigators fill in clinical forms and give psychological and QOL questionnaires to the parents to be completed at home. Parents are asked to fill PWS psychopathology questionnaire together on behalf of the Prader-Willi child and each parent is asked to fill its own psychological and QOL questionnaire. All paper forms are returned to the reference centre coordination team and entered into the database by CRA as previously described. Data entry of all study forms at inclusion (MSD, psychological and QOL) is done within one hour and a half, including intermediary CBCL scores calculation using Assessment Data Manager software. Data entry of follow-up forms is done within half an hour.

We introduced a double check procedure in order to obtain high-quality data. i) patient's data are checked by CRA at the time of data entry, using data management forms to display summary or detailed data entered in the database. These forms are written-protected to avoid unwanted data modification and can be sorted and filtered in various ways. ii) a set of calculated coherence controls is computed monthly by CRA using Stata 8.2 software as previously described.

Error correction procedure is as follows: a standardized request form is sent to the investigator to be filled in and returned to the coordination team. When the investigator answer is received, database values are corrected if needed, date of corrections and name of CRA being noted on request form. Request form is then archived with source data.

Data entry quality has been assessed prior to the statistical analysis of the transversal study (work in progress). We randomly selected 10% of patient's medical records and we compared source data with database data. Low error rate (0.3%) confirmed the quality of the tools and procedures used in this project. Further regular data quality controls are scheduled, a double data entry procedure being required if 5% error rate is exceeded.

## Utility

This database will be a powerful tool to collect data on patients presenting this complex and multi system disease and will be a basis for the design of future prospective multicentric studies. It might allow us to analyse the strengths and weaknesses of follow-up strategies used in everyday clinical practice, taking in account clinical improvements as well as patient's psychological status and parents psychological and quality of life repercussions.

Moreover this database could help sharing experience with other worldwide teams involved in management and care of PWS patients; particularly those who also set-up databases and could provide data to support the onset of a future European PWS database.

This publication could also provide landmarks for other teams having such a project and might be of help for teams interested in setting up cohort studies in other rare diseases involving some of the PWS related troubles.

## Discussion

Nowadays, PWS research is clearly active as shown by the outcome of expert's meetings (the 1st one held in Zurich in 2002 and the 2nd in Toulouse in 2006). The genetic research showed evidence about some phenotype-genotype correlations [39-42] and many clinical studies mainly based on the effects of growth hormone (GH) treatment have been published in children [43-46] and adults with PWS [47-49]. In addition, post-marketing databases [50,51] reported strong evidence for hormonal therapy benefit and behaviour management efficiency.

Most of the data and particularly history and care came from clinical studies involving a small number of patients enrolled by few highly specialized teams, leading not only to limit statistical power but also to emphasize bias related to inclusion criterion, patients' selection and team's routine clinical practice. Moreover these studies being focused on a set of clinical and/or biological variables are unable to reflect the whole patients' profile. But, on another hand, setting up a prospective study exploring interactions between clinical treatment, psychological status and familial context does not seem to be feasible, mainly due to the high number of patients needed to be included which is not compatible with a rare disease. At last, most of clinical studies are of relatively short duration and cannot reflect the long term evolution of the disease nor period-specific difficulties related to clinical management such as childhood to adult transition.

This database will be of primary interest to improve the knowledge of PWS disease: it will be a powerful tool for future specific research projects such as epidemiological studies. Main characteristics of the population at inclusion are listed in table 3. Today, two years after the beginning of the inclusions, 146 patients (81% of estimated PWS cases) have been included and none of the PWS families refused to be enrolled in the study. Therefore, we consider that making the actual database to become an exhaustive register including all eligible patients at a national level is a realistic and feasible goal that will be one of the reference centre objectives in the years to come. The follow-up of such an exhaustive cohort could allow us to improve the knowledge of the evolution of this disease, to optimize therapies and to adjust multidisciplinary care organisation, taking into account not only the PWS patients' clinical profile but also their familial environment.

**Table 3:** Characteristics of the population at inclusion

**Variables**	**Number of observations**	**Results expressed as % or median (min-max)**
Sex ratio	146	52.7% boys
Chronological age	146	7.24 years (0.21–18.75)
Genetic diagnosis	146	Deletion 63.7%
		Disomy 24%
		Translocation 1.4%
		Imprinting defect 2.0%
		AMP* 8.9%
Age at genetic diagnosis	143	2 months (5 days-12 years)
Obesity	138	43.5%
GH treatment	139	85.6%
Maternal age at birth of PWS child	137	32 years (18–46)
Paternal age at birth of PWS child	137	34 years(22–62)
Married	137	89.8%
Number of siblings (including PWS child)	144	2 (1–9)
Profession of the father	125	Full time 87.2%
		Part time 4.8%
		No 8%
Profession of the mother	130	Full time 26.1%
		Part time 28.5%
		No 45.4%
Social economic status	124	Low 31.4%
		Medium 46.8%
		High 21.8%
Membership of French PW Association	129	59%

* AMP = Abnormal Methylation Profile

In that way, we noticed that data collection has been considered of great help by PWS physicians for everyday clinical practice: transversal study questionnaires have been reported to be a helpful tool to summarize PWS medical history and yearly follow-up forms will provide an overview of the disease evolution that will help physicians to underline the most topical issues of patient follow-up and care. In that way, it can be considered as a clinical tool to be used in complement of patient's medical records.

We chose to start with paper forms centralized by a coordination centre then entered into the database by CRA rather than internet forms filled in by physicians. This organization appears to be adapted to a rare disease study. We also noted that giving life to the investigators' team through meetings, e-mail newsletters, phone calls and some further CRA travels to give help, maintained the motivation and ensured the long-term involvement of all members.

A further database development focused on adult PWS is planed for the next year in a national basis and links between the 2 databases will be developed and will allow us to describe the long term follow-up of early diagnosed patients receiving multidisciplinary care. This two-step development has been decided because relevant data to be gathered are clearly different between adults (i.e. aged more than 20) and younger PWS patients. This difference is due on one hand to the natural evolution of the disease and on the other hand to the history of PWS diagnosis and treatment: PWS genetic diagnosis became available in routine in the early 90's [52] and has been followed by major improvements in therapies, medical care and follow-up organization. Therefore, for PWS patients aged less than twenty, PWS genetic diagnosis was available in the first years of life, hormonal therapies were widely used in respect of guidelines and multi-disciplinary care of the disease became widespread. In contrast, for PWS patients aged more than twenty, PWS diagnosis has been based on clinical signs then confirmed or sometimes undermined by genetic diagnosis many years later. Hormonal therapy was not routinely prescribed and psychological and social cares were not developed enough. Therefore studying the adult PWS population requires a specific tool, adapted to the heterogeneity of the population and to the widespread differences related to psychosocial status. Improving our knowledge of the nowadays adult PWS patients' profile will help to refine specific follow-up as well as to adapt childhood to adult transition strategies.

## Conclusion

In 2007 the French ministry of health has extended the PWS reference centre area to the whole French territory, prompting us to implement the database with the patients of our whole country. This can be done without software modifications, allowing us to focus on the training of newly involved medical teams and on the study management organisation. Adult database is also in progress and a second CRA has been recruited to ensure the success of this cohort study extension.

## Availability and requirements

Complete listings of variables of the MSD and follow-up forms are available at . The complete database and the Stata.do files are available to any researcher wishing to use them for non-commercial purposes and can be provided upon request to the corresponding author. Links between this database ant the European database are in discussion.

## Competing interests

The authors from the coordination of the French reference centre (CM, GD, MG, MT) received funding from Pfizer for setting-up the PWS database. MT received consulting fees from Pfizer.

## Authors' contributions

CM was responsible for collecting and entering the data in the database. LC constructed the database and wrote the Stata programs. CM and LC wrote the first draft of the manuscript. GD was responsible for the design of the MSD form and MG for the design of the familial psychological forms. CA supervised the database construction and provided methodological expertise. MT as coordinator of the reference centre supervised all the aspects of the work.

## Pre-publication history

The pre-publication history for this paper can be accessed here:


